# Phase I study of LZM005 in patients with HER2-positive metastatic breast cancer

**DOI:** 10.1038/s41523-022-00501-2

**Published:** 2022-12-27

**Authors:** Cong Xue, Haifeng Li, Herui Yao, Ying Lin, Xin An, Meiting Chen, Riqing Huang, Lu Li, Anqi Hu, Mengqian Ni, Lulu Zhang, Wei Yang, Zhonghui Xu, Su Li, Yanxia Shi

**Affiliations:** 1 State Key Laboratory of Oncology in South China, Collaborative Innovation Center for Cancer Medicine, Sun Yat-Sen University Cancer Center, Guangzhou, 510060 P. R. China; 2 Department of Medical Oncology, Sun Yat-sen University Cancer Center, Guangzhou, 510060 P. R. China; 3 Guangdong Provincial Key Laboratory of Malignant Tumor Epigenetics and Gene Regulation, Department of Medical Oncology, Phase I Clinical Trial Centre, Sun Yat-sen Memorial Hospital of Sun Yat-sen University, Guangzhou, 510060 P. R. China; 4 Breast Disease Center, The First Affiliated Hospital, Sun Yat‐Sen University, Guangzhou, 510060 P. R. China; 5 Livzon Pharmaceutical Group Inc, No.38, Chuangye North Road, Jinwan District, Zhuhai, Guangdong P. R. China; 6 Department of Clinical Trial Center, Sun Yat-sen University Cancer Center, Guangzhou, 510060 P. R. China

**Keywords:** Phase I trials, Breast cancer, Drug safety

## Abstract

The prognosis of human epidermal growth factor receptor 2 (HER2) positive metastatic breast cancer (MBC) remained unsatisfactory currently, more anti-HER2 agents are needed. Here we report a phase I study that evaluated the safety, activity, and biomarkers of LZM005, a HER2 antibody, used as a monotherapy or in combination with trastuzumab plus docetaxel in patients with HER2-positive MBC. From October 2017 to December 2019, 34 patients received LZM005 (14 monotherapy, 20 combination therapy). No DLT was observed. The common adverse events (AEs) in phase Ia included diarrhea (21.4%), infusion reaction (21.4%), and hypertriglyceridemia (21.4%), while those in phase Ib were leukopenia (85.0%), neutropenia (75.0%), anemia (60.0%), diarrhea (60.0%), and rash/pruritus (50.0%). All AEs were manageable. In phase Ia, partial response (PR) was achieved in one case (1/14, overall response rate [ORR]: 7.1%); the disease control rate was 42.90% (6/14). In phase Ib, 11 patients (55.0%) achieved PR, and eight (40.0%) had stable disease. The ORR was 100% (6/6) in trastuzumab-naive and 35.7% (5/14) in trastuzumab-pretreated patients. Biomarker analysis showed that chromatin remodeling genes *KMT2B* and *BRWD1* were associated with better progression-free survival. LZM005 is well tolerated and shows potent activity in patients with HER2-positive MBC.

## Introduction

Breast cancer is the most common cancer in women. The human epidermal growth factor receptor 2 (HER2) positive subtype is an aggressive type of breast cancer, accounting for 15–25% of all breast cancer cases^1^. The approval of multiple anti-HER2 targeted agents such as trastuzumab has significantly improved the prognosis of these patients^2^. The emergence of new anti-HER2 targeted therapies, such as pertuzumab^3^, margetuximab^4^, lapatinib^5^, pyrotinib^6^, neratinib^7^, tucatinib^8^, trastuzumab emtansine (T-DM1)^9^, trastuzumab deruxtecan (T-DXd)^10,11^, and trastuzumab duocarmycin^12^, has resulted in great progress in the treatment of HER2+breast cancer. However, early recurrence and disease progression still occur while patients are on targeted therapy. Thus, new anti-HER2 regimens are urgently needed for this patient population.

Until now, the standard first-line therapy of HER2-positive metastatic breast cancer (MBC) has been docetaxel, trastuzumab, and pertuzumab, which have resulted in significantly longer survival than that offered by docetaxel and trastuzumab^3^. Pertuzumab binds to domain II of HER2 to inhibit the formation of dimers between HER2 and other HER receptors. The binding site does not overlap with trastuzumab; hence, pertuzumab combined with trastuzumab has higher efficacy^13^. Besides being a first-line regimen, pertuzumab has become a part of the standard of care in adjuvant and neoadjuvant treatment of HER2+breast cancer^14,15^. For patients who experienced progression, antibody-drug conjugates (ADCs) like T-DXd and T-DM1 were recommend^11,16,17^. However, those agents were not widely available in China leading to limited options for HER2-positive MBC, therefore, novel and easily accessed anti-HER2 agents were still needed.

LZM005 is a potent anti-HER2 antibody, which is produced by recombinant DNA technology using CHO-K1 Chinese hamster ovary cells. Compared to pertuzumab, it has a unique amino acid sequence that irreversibly binds to a different epitope of domain II of HER2 to inhibit the dimerization of HER2 with HER receptors. Its degree of humanization, stability, and affinity for HER2, and homogeneity of quality are better than those of pertuzumab. Preclinical data suggest that LZM005 can irreversibly inhibit HER2 and effectively inhibit the proliferation of HER2-overexpressing cells both in vivo and in vitro (See Supplementary Fig. 1).

Here, we conducted the first-in-human clinical trial to evaluate the safety, tolerability, and activity of LZM005 and evaluated the combination of LZM005, trastuzumab, and docetaxel as well as performed a biomarker analysis to determine the predictors of LZM005 efficacy in patients with HER2-positive MBC.

## Results

### Patients

Baseline characteristics of the patients in phase Ia and Ib are listed in Table 1. In phase Ia, 14 female patients (median age, 46.5 years; range, 29 to 67 years) were enrolled from October 2017 to March 2018. Ten patients (71.4%) received more than three lines of chemotherapy regimens in the metastatic period. Twelve (85.7%) received prior trastuzumab treatment, including three patients in the adjuvant setting and 12 in the metastatic setting. Ten patients (71.4%) presented with visceral metastasis, and five (35.7%) had liver metastasis.

**Table 1:** Patients’ baseline characteristics.

Characteristics	Ia													Ib						
	5 mg/kg (*n* = 1)			10 mg/kg (*n* = 4)		15 mg/kg (*n* = 3)		20 mg/kg (*n* = 6)			Total (*n* = 14)			420 mg (*n* = 10)		525 mg (*n* = 10)		Total (*n* = 20)		
	No.	%		No.	%	No.	%	No.		%	No.		%	No.	%	No.	%	No.		%
Median age, years (range)	56			39 (29–53)		45 (44–60)		46.5 (42–68)			46.5 (29–68)			52 (45–69)		51 (33–59)		52 (33–69)		
HR positive	0		0	3	75	0	0	3	50		6	42.9		8	80	6	60	14	70	
Median metastatic organs (range)	4			2.5 (2-3)		4 (3-4)		2 (2-5)			3 (2-5)			2 (1-4)		2 (1-3)		2 (1-4)		
Median No. of prior metastatic anticancer regimens (range)	4			5.5 (2-6)		3 (1-3)		4 (2-6)			4 (1-6)			0.5 (0-3)		1 (0-5)		1 (0-5)		
Prior anthracycline treatment	1		100	2	50	1	33.3	6	100		10	71.4		8	80	8	80	16	80	
Adjuvant	0		0	1	25	1	33.3	5	83.3		7	50		8	80	8	80	16	80	
Advanced	1		100	1	25	0	0	1	16.7		3	21.4		0	0	0	0	0	0	
Prior taxane treatment	1		100	4	100	3	100	5	83.3		13	92.9		9	90	9	90	18	90	
Adjuvant	0		0	1	25	1	33.3	4	66.7		6	42.9		8	80	9	90	17	85	
Advanced	1		100	3	75	2	66.7	5	83.3		11	78.6		2	20	0	0	2	10	
Prior trastuzumab therapy	1		100	4	100	3	100	4^a^	66.7		12	85.7		6^b^	60	8^c^	80	14	70	
Prior endocrine therapy (advanced stage)	0		0	2	50	0	0	3	50		5	35.7		0	0	1	10	1	5	

^a^In phase Ia study, there were two patients did not receive trastuzumab in advanced treatment because of economy reason.^b^In phase Ib study 420 mg arm, four patients were initially diagnosed advanced disease so they did not receive trastuzumab before.^c^In phase Ib study 525 mg arm, two patients were initially diagnosed advanced disease so they did not receive trastuzumab before.

In phase Ib, 20 female patients (median age, 52 years; range, 33 to 69 years) were enrolled from Sep 2019 to Dec 2019 at three centers. They had fewer metastatic organs and had not been heavily treated before. The mean number of previous chemotherapy regimens in the metastatic period was 1 (range, 0–5). Fourteen (70.0%) received prior trastuzumab treatment, including seven patients in the adjuvant setting, and 13 in the metastatic setting. Sixteen patients (80.0%) showed visceral metastasis, and eight (40.0%) showed liver metastasis.

### Safety

During phase Ia dose escalation, none of the patients in any of the dose cohorts experienced a DLT within 21 days of dosing. In the two arms of phase Ib, none of the patients experienced an LZM005-DLT within 21 days of dosing. Both LZM005 1050–525 mg and LZM005 840–420 mg cohorts were considered candidate dosages for the subsequent phase II study.

In phase Ia, 14/14 (100%) patients completed the first dosage treatment. The median duration of treatment was 2.5 cycles (range, 1 to 20 cycles). The median relative dose intensity was 100% for each dose level. Ten patients (71.4%) experienced LZM005-related AEs. The common AEs in the phase Ia trial included diarrhea (21.4%), infusion reaction (21.4%), and hypertriglyceridemia (21.4%). One patient experienced grade 3 anemia, which was considered to be disease-related (Table 2).

**Table 2:** Treatment-related AE in phase Ia.

	5 mg/kg (*n* = 1)		10 mg/kg (*n* = 4)		15 mg/kg (*n* = 3)		20 mg/kg (*n* = 6)		Total (*n* = 14)	
	Any grade, *n* (%)	Grade 3/4, *n* (%)	Any grade, *n* (%)	Grade 3/4, *n* (%)	Any grade, *n* (%)	Grade 3/4, *n* (%)	Any grade, *n* (%)	Grade 3/4, *n* (%)	Any grade, *n* (%)	Grade 3/4, *n* (%)
Diarrhea	0	0	1 (7.1)	0	0	0	2 (14.3)	0	3 (21.4)	0
Infusion reaction	0	0	1 (7.1)	0	1 (7.1)	0	1 (7.1)	0	3 (21.4)	0
Hypertriglyceridemia	0	0	1 (7.1)	0	1 (7.1)	0	1 (7.1)	0	3 (21.4)	0
Anemia	0	0	0	0	1 (7.1)	0	1 (7.1)	1 (7.1)	2 (14.3)	1 (7.1)
Fatigue	0	0	0	0	0	0	2 (14.3)	0	2 (14.3)	0
ALT elevation	0	0	1 (7.1)	0	0	0	1 (7.1)	0	2 (14.3)	0
Chest discomfort	0	0	2 (14.3)	0	0	0	0	0	2 (14.3)	0

In phase Ib, 20/20 (100%) patients completed the first dosage treatment. The median duration of LZM005 and trastuzumab treatment was 11 cycles (range, 2 to 24 cycles). The median duration of docetaxel treatment was 7.5 cycles (range, 2 to 9 cycles). Two patients terminated docetaxel in cycle 8 for grade 3 neurotoxicity and grade 3 edema. One patient underwent dosage reduction of docetaxel from cycle 2 for neutropenia with fever. The relative dose intensity was 100%, 100%, and 94.4% for LZM005, trastuzumab, and docetaxel. All patients experienced at least one treatment-related AE (Table 3). The most common any grade AEs in the phase Ib trial included leukopenia (85.0%; 420 mg arm, 90%; 525 mg arm, 80%), neutropenia (75.0%; 420 mg arm, 90%; 525 mg arm, 60%), anemia (60.0%; 420 mg arm, 70%; 525 mg arm, 50%), diarrhea (60.0%; 420 mg arm, 60%; 525 mg arm, 60%), and rash/pruritus (50.0%; 420 mg arm, 70%; 525 mg arm, 30%). Grade 3 treatment-related AEs occurred in 16 (80.0%; 420 mg arm, 90%; 525 mg arm, 70%) patients, including neutropenia (70.0%; 420 mg arm, 90%; 525 mg arm, 50%) and leukopenia (55.0%; 420 mg arm, 70%; 525 mg arm, 40%). No treatment-related death was observed. Notably, no cardiovascular AEs were reported in the phase Ia and Ib studies.

**Table 3:** Treatment-related AE in phase Ib.

	LZM005 420 mg+TH (*n* = 10)		LZM005 525 mg+TH (*n* = 10)		Total (*n* = 20)	
	Any grade, *n* (%)	Grade 3/4, *n* (%)	Any grade, *n* (%)	Grade 3/4, *n* (%)	Any grade, *n* (%)	Grade 3/4, *n* (%)
Leukopenia	9 (90)	7 (70)	8 (80)	4 (40)	17 (85)	11 (55)
Neutropenia	9 (90)	9 (90)	6 (60)	5 (50)	15 (75)	14 (70)
Anemia	7 (70)	1 (10)	5 (50)	0	12 (60)	1 (5)
Diarrhea	6 (60)	0	6 (60)	1 (10)	12 (60)	1 (5)
Rash/ pruritus	7 (70)	0	3 (30)	0	10 (50)	0
Fatigue	6 (60)	0	2 (20)	0	8 (40)	0
Infusion reaction	4 (40)	0	4 (40)	0	8 (40)	0
Neurotoxicity	6 (60)	0	2 (20)	0	8 (40)	0
Hand-Foot-Syndrome	0	0	1 (10)	1 (10)	1 (5)	1 (5)
Gastrointestinal bleeding	1 (10)	1 (10)	0	0	1 (5)	1 (5)
Dyspnea	1 (10)	0	1 (10)	1 (10)	2 (10)	1 (5)
Lymphopenia	1 (10)	1 (10)	0	0	1 (5)	1 (5)

No antibodies to LZM005 were detected.

### Serum pharmacokinetics

#### Phase Ia

**Fig. 1:**
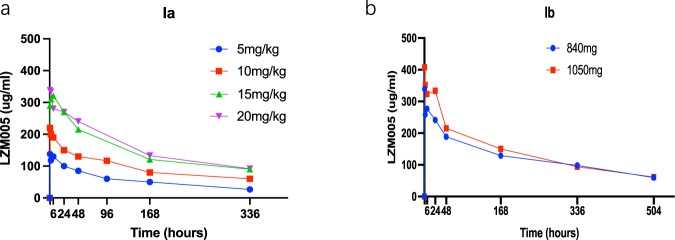
The Serum Pharmacokinetics profiles of LZM005. **a** The serum concentration–time profiles of phase Ia; **b** the result serum concentration–time profiles of phase Ib. concentration–time profiles of phase Ib.

The pharmacokinetic parameters estimated by POWER modeling were summarized in Table 4. The mean LZM005 concentration versus time curves during cycles 1 at designed time points were shown in Fig. 1a. And mean serum concentrations were maintained above 20 ug/ml fortnight from first administrated. At LZM005 dose of 20 mg/kg of phase Ia, the serum concentrations were steadily maintained at a level around 150 ug/ml since cycle 4. The AUC_0-t_ and C_max_ were increased statistically with the dose escalated and stable at the dose of 15 mg/kg but failed to exhibit a significant linear relationship in the designed dose range (r^¼^ 0.71 and r^¼^ 0.68, respectively). Systemic clearance, volume of distribution at steady-state, and the elimination half-life did not change with dose.

**Table 4:** LZM005 Pharmacokinetic Parameter Estimates in Phase Ia.

	CL (ml/h)		Vss (ml)		AUC_0-t_ (h*mg/ml)		Cmax (ug/ml)		*t*_1/2_ (h)	
Dose Group(mg/kg)	Mean	SD	Mean	SD	Mean	SD	Mean	SD	Mean	SD
5 mg/kg (*N* = 1)	10.433		3382.474		22.546		132.385		211.122	
10 mg/kg(*N* = 4)	8.989	2.118	4508.610	430.952	36.995	6.842	219.402	39.061	374.983	63.231
15 mg/kg(*N* = 3)	11.576	3.8161	3708.281	354.665	57.221	4.925	345.502	36.801	244.263	57.010
20 mg/kg(*N* = 6)	16.279	5.464	4609.199	599.711	60.257	13.253	340.037	40.567	215.294	72.651

#### Phase Ib

Due to the quality of sampling, five and eight patients were included into final analysis for 840 mg and 1050 mg group, respectively. The mean LZM005 concentration versus time curves during cycles 1 at designed time point of two doses were shown in Fig. 1b. The pharmacokinetic parameters were summarized at Table 5. Briefly, the Cmax (345.5 ug/ml vs 432.1 ug/ml) and AUC_0-24h_ (61.8 hr*mg/ml vs 77.9 hr*mg/ml) were increased as the dose escalated while the systemic clearance, half-life and trough concentrations were comparable. And the serum concentrations were stable since second administrated.

**Table 5:** LZM005 Pharmacokinetic Parameter Estimates in Phase Ib.

	CL (ml/h)		Vz (ml)		AUC_0-24h_ (h*mg/ml)		Cmax (ug/ml)		*t*_1/2_ (h)	
Dose group	Mean	SD	Mean	SD	Mean	SD	Mean	SD	Mean	SD
840 mg (*N* = 5)	13.370	6.245	3768.000	1219.000	61.820	15.000	345.500	77.920	202.600	31.460
1050 mg (N = 8)	13.170	0.533	3458.000	1110.000	77.900	7.584	432.100	98.120	183.300	65.840

### Antitumor activity

**Fig. 2:**
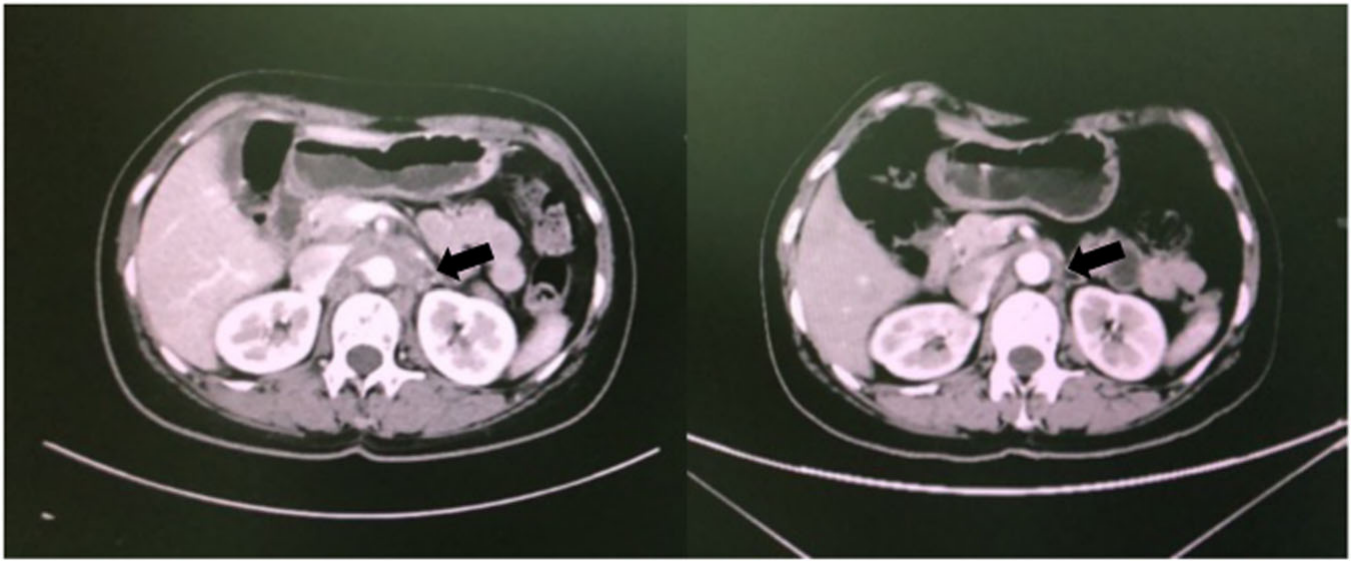
The efficacy of LZM005 in a phase Ia patient. Significant target lesion response (PR) in a patient with LZM005 20 mg/kg in CT scan (black arrow indicated the lesion, left: pre-treated, right: post-treated).

In the phase Ia study, one patient achieved PR (20 mg/kg), six showed stable disease (SD), and seven showed progressive disease (PD). The ORR was 7.1%, and the DCR was 42.9%. Median PFS was 1.37 months (95% CI, 1.31 to 1.47 months). Median OS was 10.73 months (95% CI, 9.37 to 12.09 months). Among the six patients with SD, five continued to show SD for more than 12 weeks. The median duration of SD was 4.07 months (95% CI, 3.43 to 4.71 months). For the patient with PR, the duration of response was 2.83 months (Fig. 2). At the time of data cutoff (December 31, 2020), all patients showed disease progression.

**Fig. 3:**
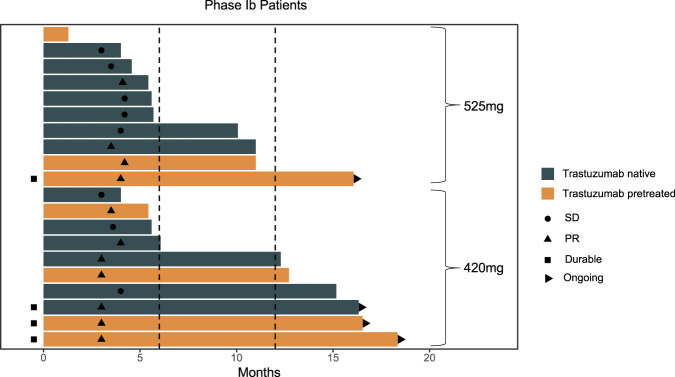
The swimmer plot of patient in Phase Ib. The progression-free survival of LZM005 for patients in arm 525 mg and arm 420 mg.

In the phase Ib study, 11 patients (55.0%) achieved PR, eight (40.0%) had SD, and one showed PD. The ORR was 100% (6/6) in trastuzumab-naïve patients and 35.7% (5/14) in trastuzumab-pretreated patients. DCR was 92.9% (13/14) in trastuzumab-pretreated patients. The ORR and DCR were respectively 70.0% and 100% for the 420 mg arm, and 40.0% and 90.0% for the 525 mg arm (Fig. 3). Median PFS was 6.1 months (95% CI, 0 to 15.74 months) with a median follow-up time of 16.43 months (95% CI, 16.16 month to not reached). Median OS could not be determined due to the limited number of events. Among the eight patients with SD, all showed SD for more than 12 weeks. The median duration of SD was 10.13 months (95% CI, 4.45 to 15.82 months). For the 11 patients showing PR, the median duration of response was 9.87 months (95% CI, 7.75 to 11.98 months). At the time of data cutoff (December 31, 2020), four patients had ongoing LZM005 and trastuzumab treatment.

The PFS of seven patients who received first-line therapy was not reached (four of them were on the maintenance treatment). For the six patients who received second-line therapy, the PFS was 5.63 months (95% CI, 5.44 to 5.82 months). For the remaining seven patients with more than third-line therapy, the PFS was 5.73 months (95% CI, 1.37 to 10.10 months).

### Biomarker analyses

For the genomic biomarker studies, we generated WES data from 18 tumor tissue samples. In the present genomic analysis, seven patients with PFS longer than 6 months were classified as good responders and 11 patients with PFS shorter than 6 months were classified as poor responders.

**Fig. 4:**
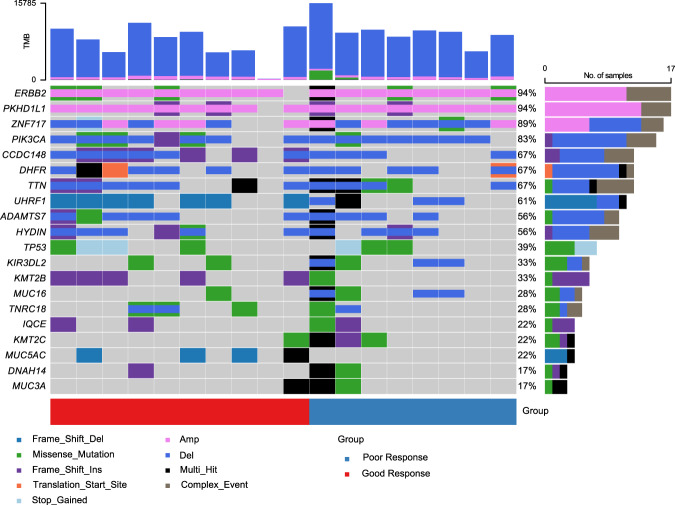
The overview of genomic alterations. The oncoplot of 18 patients in Phase Ib.

The landscape oncoplot of copy number alterations and non-synonymous mutations is presented in Fig. 4. In the subsequent profiling, we found that eight genes (*BRWD1, MELK, MIR4475, PCNT, RNF38, ASPA, TRPV1*, and *TRPV3*) had different alteration trends between groups (all *p* < 0.05). (Supplementary Table 1) However, after adjustment for multiple comparisons, no differentially mutated genes were identified between the two groups. No significant preexisting alternative onco-pathway was found in the present analysis. With regard to the PFS impact of genomic alterations, we found that *KMT2B, MUC16*, and *KIR3DL2* were associated with PFS in the univariate Cox model thought failed to meet the statistical boundary in multivariate model. Thus, patients with *KMT2B* mutations had a longer progression-free interval (NR vs. 5.67 months, HR 0.2, *p* = 0.045) than their non-alters while patients with *MUC16* (5.63 months vs. 11.10 months, HR 3.84, *p* = 0.035) or *KIR3DL2* (5.67 months vs. 13.17 months, HR 4.0, *p* = 0.033) mutations showed faster progression than their counterparts. Notably, among the six patients (6/18, 33%) who harbored a *KMT2B* mutation, five belonged to the good responder group and all were identified as having a frameshift ins mutation.

## Discussion

In the present study, we first assessed the tolerability and efficacy of LZM005 in HER2+MBC in a phase I trial, including when it was used as monotherapy and added to trastuzumab plus docetaxel. We also performed accompanying genomic profiling to explore the potential biomarkers of LZM005-based treatment. In the phase Ia study, no DLT was observed, and the ORR was 7.1% while the DCR was 42.9%. In the phase Ib study, we achieved an ORR of 55% with a median PFS of 6.1 months and tolerable toxicity that was consistent with previous studies on HER2+MBC combination therapy^13^. In the WES profiling, we found that the epigenetic genes *KMT2B* and *BRWD1* might be associated with treatment outcomes and that *MUC16* and *KIR3DL2* mutations may have potential effects on the PFS.

In phase Ia, the most common any grade AE’s included diarrhea, infusion reaction, hypertriglyceridemia, fatigue, anemia, ALT elevation, and chest discomfort. And one patient experienced grade 3 anemia. All AEs were modest and manageable, in line with the findings of previous studies on pertuzumab^13^. We observed more AEs in combination regimen than in monotherapy, but the AEs, including leukopenia, neutropenia, and anemia, were more related to docetaxel than the antibodies^3^. Grade 3 AEs necessitated a docetaxel dosage reduction in one patient and termination of docetaxel in two patients. No LZM005-related SAEs were observed. Diarrhea was commonly seen in patients treated with LZM005, trastuzumab and docetaxel, and we assumed the frequency and degree of diarrhea were increased in the combination therapy. No unplanned AEs and SAEs were observed. In comparison with the combination of pertuzumab, trastuzumab, and docetaxel, our combination showed similar AEs^3^. Cardiotoxicity was not observed in our study. The findings suggested that LZM005 had better safety compared to pertuzumab.

Although multiple HER2-directed agents have become available in recent years, the preferred option for HER2+MBC remains taxane-based chemotherapy combined with dual anti-HER2 antibodies, especially for treatment-native patients. The evidence of such a regimen came from the CLEOPATRA trial, which showed that the combination of trastuzumab plus pertuzumab plus a taxane yielded an 80% ORR and a 7-month PFS advantage when compared to trastuzumab plus a taxane^3^. Unfortunately, this option is not included in our public health insurance program for the HER2 + MBC yet as well as T-DM1^9^ which usually means unbearable prices for common patients. Although T-Dxd^11^ and tucatinib are also serviced as alternatives after progression, they are troubled by the availability issue currently in China. Additionally, there is no consensus regarding the preferred therapeutic strategy for HER2+MBC patients who required later-line treatment^18^. A phase II study focused on the efficacy of dual anti-HER2 antibodies combined with another mitotic block agent, eribulin, also achieved 17.8% ORR and 6 months PFS^19^. In the present study, we achieved 100% ORR in trastuzumab-naive patients and 95% DCR in the total cohort in the combination therapy phase. These data suggested that the LZM005 is a potent alternative for HER2+MBC.

In recent years, numerous clinical trials have demonstrated that dual-anti-HER2-based regimens had considerable efficacy for HER2+MBC. However, patients who harbored certain efficacy-related genomic alterations as a result of previous heavy treatment still had a low response rate^20^. Therefore, tailored regimens and expected benefit assessment on the basis of genomic mutations played an increasingly important role in personalized treatment^18^. In the present study, we identified eight candidate genes that may affect efficacy by using a 6-month PFS interval. For example, *MELK* had been proven to promote the proliferation in TNBC and ER+cell lines via arrest of cell cycles^21^, although its function in pure HER2+breast cancer is not fully understood. Moreover, the candidate genes *TRPV1* and *TRPV3*, from the *TRPV* channel family, have been reported to be potential therapeutic targets in breast cancer^22^. As noted in our study, patients with *ASPA* mutations had a worse outcome with LZM005-based therapy^23^, however, we thought that these patients were non-responsive for docetaxel instead of HER2 antibodies since *ASPA* was a microtubule-associated protein. Interestingly, we had identified two chromatin remodeling genes that might influence the efficacy of dual-anti-HER2- based therapy, *KMT2B* and *BRWD1. KMT2B*, one of the genes in the *KMT2* family, controlled the trimethylation of histone H3 lysine4 (H3K4me3) sequenced to activate the transcription of downstream genes^24–27^. In a previous in vitro study, a member of *KMT2* family, *KMT2D*, was reported to be a regulator that influenced the sensitivity of lapatinib in HER2+breast cancer cell lines^28^. Similar to our study, patients with a *KMT2B* frameshift mutation had a significant PFS advantage over their counterparts, which suggested that the trimethylation of H3K4 may play an important role in anti-HER2 therapies. In addition, in our study, the histone lysine-acetylation reader *BRWD1*^29^, a member of the WD40 repeat protein families that associates with the SWI/SNF complex, had a disproportional mutation tendency in the poor response group. These data imply that the epigenetic process impaired the efficacy of dual-anti-HER2-based therapy in certain cases, and the addition of epigenetic inhibitors to the conventional regimen may yield more benefits for HER2+MBC.

Nevertheless, this study had various limitations. First, the small sample size may have compromised the true efficacy of LZM005, and obscured the influence of genomic modalities on LZM005-based treatments. Additionally, while samples were collected before treatment, the event-based samples should be taken into consideration to verify the impact of genomic mutations on the LZM005-based treatment. In phase Ib, we observed a satisfactory treatment response in trastuzumab-naïve patients though it might be compromised by the limited size. Therefore, further head-to-head randomized phase III studies compared with pertuzumab are warranted to confirm the efficacy and safety of LZM005 in patients with HER2-positive MBC.

In summary, LZM005 is well tolerated and safe with potent efficacy for patients with HER2+MBC. Genomic alterations of the chromatin remodeling genes *KMT2B* and *BRWD1* may play important roles in the outcomes of LZM005-based therapy.

## Methods

### Study design

This was an open-label, first-in-human phase I study. The study included phases Ia and Ib. Phase Ia had a single-center, dosage escalation design. LZM005 monotherapy was administered intravenously at doses of 5, 10, 15, and 20 mg/kg. The primary endpoints were dose-limited toxicity (DLT), maximum-tolerated dose (MTD), safety, and tolerability. The secondary endpoints were pharmacokinetics (PK), immunogenicity, and efficacy. In phase Ib, LZM005 was administered with trastuzumab and docetaxel at the MTD. The primary endpoints included safety, tolerability, and the dose for further studies. The secondary endpoints were PK, immunogenicity response, and biomarker analysis. The study was conducted in accordance with the International Conference on Harmonization Guideline for Good Clinical Practice and the Declaration of Helsinki. The study protocol was approved by the institutional review boards of Sun Yat-Sen University Cancer Center, Sun Yat-Sen Memorial Hospital (S.Y.S.M.H.), and Sun Yat-Sen First Affiliated Hospital (S.Y.S.F.A.H.; S.Y.S.M.H. and S.Y.S.F.A.H. participated in the phase Ib study). Written informed consent was obtained from all patients before their enrollment in this study. The clinical trial numbers were CTR20170991 (phase Ia, 2017-09-11) and CTR20191921 (phase Ib, 2019-09-29), and the trials were registered in www.chinadrugtrials.org.cn.

The efficacy evaluation was performed in accordance with RECIST 1.1^30^. Responses were evaluated every two cycles. The efficacy evaluation included the overall response rate (the percentage of complete response [CR] + partial response [PR]), duration of response (from the first time of CR/PR estimation to the date of progression or death), disease control rate (the percentage of confirmed CR + PR + stable disease [SD]), progression-free survival (PFS, from the date treatment began to the date of progression or death), and overall survival (OS, from the date treatment began to the date of death).

### Patient eligibility

Patients who satisfied the following inclusion criteria were eligible for phases Ia and Ib: 1) age between 18 and 70 years, 2) a histologic diagnosis of MBC with a positive HER2 status (immunohistochemistry 3+ , or 2+ confirmed by fluorescence in situ hybridization [FISH]). Hormone receptor status should be recorded but unrestricted. 3) an Eastern Cooperative Oncology Group [ECOG] score of 0 to 1, 4) presence of at least one measurable lesion according to the RECIST 1.1 criteria, 5) nonavailability of standard treatments or unwillingness to receive standard treatment, and 6) adequate bone marrow and organ function, 7) left ventricular ejection fraction (LVEF) of 50% or more at baseline (determined by echocardiogram). There was no limit on the number of prior therapies. However, patients were excluded if they had received prior treatment with pertuzumab.

### Dose escalation

In phase Ia, the dose-escalation part used two consecutive phase I designs. Firstly, the study began with an accelerated titration design (ATD) on the first dosage 5 mg/kg for one patient. Then it transitioned to a traditional 3 plus 3 dose-escalation design. The ATD was used to limit the number of patients treated at potentially subtherapeutic doses during the phase I study. Escalation dosing included the administration of 10, 15, and 20 mg/kg doses intravenously once in 21-day cycles. The dosage arm was set referring to the safety and tolerability data of pertuzumab in Asian patients. No MTD was reached in pertuzumab with 5–20 mg/kg. If no patient experienced a DLT in the first cycle, three patients were enrolled to the next dosage level. If a patient experienced a DLT, then additional three patients were enrolled at the same dosage level. If 2 out of six patients at a dosage level experienced a DLT, dose escalation was stopped and the previous dosage level was considered the MTD. In the 20 mg/kg arm, if no DLT was observed in the first three patients, another 3–6 patients were enrolled in this dosage arm for further safety and efficacy evaluation. The first infusion time was 90 ± 10 min; all subsequent infusions were 60 ± 10 min if no infusion reaction occurred the first time.

According to the National Cancer Institute Common Terminology Criteria for Adverse Events version 4.0, a DLT was defined as any LZM005-related AE of hematologic grade 4 persisting for more than 7 days, grade 3 neutropenia with fever (body temperature of ≥38.5 °C), grade 4 thrombocytopenia with bleeding tendency, grade 4 anemia, grade 4 infusion reaction, or other nonhematologic AE of grade 3 or 4 persisting for more than 3 days (besides alopecia). Cardiac function (reported as LVEF) was monitored at baseline and every 6 weeks using echocardiogram. Additionally, electrocardiographs and serum markers of cardiac damage (troponin T) were collected every cycle.

In phase Ib, two dosage levels of LZM005 were set: 1) 840 mg loading dose---420 mg maintenance dose, and 2) 1050 mg loading dose---525 mg maintenance dose. The dosage setting was performed with pertuzumab (840 mg–420 mg) as the reference, and the dose was increased by 25% for further evaluation. Trastuzumab was administered with an 8 mg/kg loading dose and 6 mg/kg maintenance dose and an interval of 21 days. Docetaxel was administered at 75 mg/m^2^ with an interval of 21 days. The order of administration was LZM005, trastuzumab, and docetaxel. The interval between the administration of two agents was 1 h ± 3 min. Docetaxel was administered for eight cycles, except for that in patients with grade 3 or 4 AEs. More cycles were administered if the patients tolerated the treatment. G-CSF was not used in cycle 1 for prophylaxis of febrile neutropenia. If more than grade 3 leukopenia happened in cycle 1, G-CSF could be used after 24 to 48 h of chemotherapy. Trastuzumab and LZM005 were administered continually until disease progression or the development of intolerable toxicity. There was no dosage reduction for trastuzumab and LZM005. The docetaxel dose was reduced to 60 mg/m^2^ if grade 4 neutropenia persisted for more than 7 days or neutropenia with a fever of over 38.5 °C developed. Docetaxel was terminated in the following cases: (1) severe allergy, (2) grade 3 or 4 neuropathy, or (3) a skin reaction that did not resolve after dose reduction.

### Safety

Safety evaluations included the assessment of vital signs and medical history, physical and laboratory examinations, electrocardiograph, and echocardiogram. In phase Ia, safety evaluations were conducted on days 1, 2, 3, 5, 8, and 15 of cycle 1; day 1 after cycle 2; and day 28 at the end of treatment. In phase Ib, safety evaluations were conducted on days 1, 2, 3, 8, and 15 of cycle 1; day 1 of cycles 2 to 3; day 1 of every three cycles after cycle 4; and day 28 at the end of treatment. Follow-up evaluations to determine survival were conducted every 3 months.

### Pharmacokinetics analysis

Pharmacokinetic parameters were estimated from LZM005 concentrations measured using serum samples taken at specified time points postdosing. In treatment cycle 1, samples were taken before treatment, at 0.5, 2 and 6 h after the completion of the first infusion (day 1), and on days 2, 3, 5, 8, and 15. For treatment cycle 2 and beyond, samples were taken before infusion, 0.5 h after the completion of infusion, and on the end of trial. Concentrations of LZM005 were determined by a receptor-binding, enzyme-linked, immunosorbent assay (ELISA). The assay used p185HER-2 extracellular domain to capture LZM005 from serum samples. Bound LZM005 was detected with mouse antihuman Fc-horseradish peroxidase (Jackson Immuno Research Laboratories Inc, West Grove, PA), and tetramethyl benzidine (KPL Inc, Gaithersburg, MD) was used as the substrate for color development to quantify serum LZM005 against a known standard curve. The assay can detect a minimum quantifiable concentration of 0.25 ug/mL for LZM005 in human serum. The assay has <20% coefficient of variation for inter-assay and intra-assay variability, and serum spike recoveries of LZM005 between 80% and 120%.

### Immunogenicity analysis

Serum samples, taken before first infusion and at the last study visit, were tested for anti-LZM005 antibody titers using a bridging ELISA. The assay used LZM005 to capture anti-LZM005 antibodies, and then the presence of bound anti-LZM005 anti- bodies were detected with biotinylated LZM005 and streptavidin horseradish peroxidase, using tetramethyl benzidine as a substrate for color development. An antibody titer of >2.0 was considered positive.

### Biomarker analysis

Pretreatment formalin-fixed paraffin-embedded tumor samples with matched normal tissue/blood samples were obtained and used for whole-exome sequencing (WES). WES data were processed using published pipelines. After quality-control exclusions, the final cohort for WES analysis included 18 patients in phase Ib. Sequencing libraries were built using the Agilent Sure Select Human All Exon V6 Kit (Agilent Technologies, CA, USA) and then sequenced on an Illumina HiSeq platform. Reads were aligned to the reference genome (hg19) by using bwa and samblaster, and soft-clipped reads were filtered. Somatic mutation analysis was performed with Mutect2^31,32^. Copy number variations were performed with GISTIC2^33^. Tumor mutational burden was calculated as the number of detected mutations over the region of a tumor genome as previously described^34^. To explore the associations between genomic alterations and clinical benefits, the patients were regrouped according to the duration of clinical benefit (DCB). Patients whose DCB persisted longer than 6 months were identified as good responders, while the counterparts were classified as poor responders.

### Statistical analysis

Nonparametric statistical tests were used to compare the groups, and Fisher’s exact tests were used for binary data comparisons. Mann–Whitney U tests were used to compare continuous data. All statistical analyses were performed with R software (Version 3.6.0). A *p* value of less than 0.05 was considered statistically significant, and all *P* values were two-tailed.

## Supplementary information

reporting-summary

supplementary information

## Data Availability

The raw sequencing data were deposited at Sequence Read Archive database (PRJNA898328). Other data generated in this study were deposited at Research Data Deposit (https://www.researchdata.org.cn/, RDDA2022952814). If a researcher wants to use our raw data for scientific research purposes, he or she could apply for use with our corresponding author and database administrator.

## References

[1] Slamon DJ (2001). Use of chemotherapy plus a monoclonal antibody against HER2 for metastatic breast cancer that overexpresses HER2. N. Engl. J. Med..

[2] Ligibel JA, Winer EP (2002). Trastuzumab/chemotherapy combinations in metastatic breast cancer. Semin Oncol..

[3] Swain SM (2015). Pertuzumab, trastuzumab, and docetaxel in HER2-positive metastatic breast cancer. N. Engl. J. Med..

[4] Rugo HS (2021). Efficacy of Margetuximab vs Trastuzumab in Patients With Pretreated ERBB2-Positive Advanced Breast Cancer: A Phase 3 Randomized Clinical Trial. JAMA Oncol..

[5] Geyer CE (2006). Lapatinib plus capecitabine for HER2-positive advanced breast cancer. N. Engl. J. Med..

[6] Xu B (2021). Pyrotinib plus capecitabine versus lapatinib plus capecitabine for the treatment of HER2-positive metastatic breast cancer (PHOEBE): a multicentre, open-label, randomised, controlled, phase 3 trial. Lancet Oncol..

[7] Saura C (2014). Safety and efficacy of neratinib in combination with capecitabine in patients with metastatic human epidermal growth factor receptor 2-positive breast cancer. J. Clin. Oncol..

[8] Murthy, R, K. et al. Tucatinib, Trastuzumab, and Capecitabine for HER2-Positive Metastatic Breast Cancer. *N. Engl. J. Med.* **382**, 597–609 (2020). 10.1056/NEJMoa1914609 31825569

[9] Verma S (2012). Trastuzumab emtansine for HER2-positive advanced breast cancer. N. Engl. J. Med..

[10] Modi S (2020). Trastuzumab Deruxtecan in Previously Treated HER2-Positive Breast Cancer. N. Engl. J. Med..

[11] Cortes J (2022). Trastuzumab Deruxtecan versus Trastuzumab Emtansine for Breast Cancer. N. Engl. J. Med..

[12] Banerji U (2019). Trastuzumab duocarmazine in locally advanced and metastatic solid tumours and HER2-expressing breast cancer: a phase 1 dose-escalation and dose-expansion study. Lancet Oncol..

[13] Agus DB (2005). Phase I clinical study of pertuzumab, a novel HER dimerization inhibitor, in patients with advanced cancer. J. Clin. Oncol..

[14] Gianni L (2016). 5-year analysis of neoadjuvant pertuzumab and trastuzumab in patients with locally advanced, inflammatory, or early-stage HER2-positive breast cancer (NeoSphere): a multicentre, open-label, phase 2 randomised trial. Lancet Oncol..

[15] Piccart M (2021). Adjuvant Pertuzumab and Trastuzumab in Early HER2-Positive Breast Cancer in the APHINITY Trial: 6 Years’ Follow-Up. J. Clin. Oncol..

[16] Pegram MD (2004). Results of two open-label, multicenter phase II studies of docetaxel, platinum salts, and trastuzumab in HER2-positive advanced breast cancer. J. Natl Cancer Inst..

[17] Robert N (2006). Randomized phase III study of trastuzumab, paclitaxel, and carboplatin compared with trastuzumab and paclitaxel in women with HER-2-overexpressing metastatic breast cancer. J. Clin. Oncol..

[18] Martinez-Saez O, Prat A (2021). Current and Future Management of HER2-Positive Metastatic Breast Cancer. JCO Oncol. Pr..

[19] Balch SM (2021). A phase II study of efficacy, toxicity, and the potential impact of genomic alterations on response to eribulin mesylate in combination with trastuzumab and pertuzumab in women with human epidermal growth factor receptor 2 (HER2)+ metastatic breast cancer. Breast Cancer Res Treat..

[20] Liu X (2021). Next-generation sequencing revealed recurrent ZFPM1 mutations in encapsulated papillary carcinoma of the breast. NPJ Precis Oncol..

[21] Li G (2018). MELK as a potential target to control cell proliferation in triple-negative breast cancer MDA-MB-231 cells. Oncol. Lett..

[22] Lozano C (2018). Intracellular aggregated TRPV1 is associated with lower survival in breast cancer patients. Breast Cancer. (Dove Med Press).

[23] Rouquier S (2014). Expression of the microtubule-associated protein MAP9/ASAP and its partners AURKA and PLK1 in colorectal and breast cancers. Dis. Markers..

[24] Alam H (2020). KMT2D Deficiency Impairs Super-Enhancers to Confer a Glycolytic Vulnerability in Lung Cancer. Cancer Cell..

[25] Ladopoulos V (2013). The histone methyltransferase KMT2B is required for RNA polymerase II association and protection from DNA methylation at the MagohB CpG island promoter. Mol. Cell Biol..

[26] Rao RC, Dou Y (2015). Hijacked in cancer: the KMT2 (MLL) family of methyltransferases. Nat. Rev. Cancer..

[27] Su CH (2016). Regulation of IL-20 Expression by Estradiol through KMT2B-Mediated Epigenetic Modification. PLoS One..

[28] Matkar S (2015). An Epigenetic Pathway Regulates Sensitivity of Breast Cancer Cells to HER2 Inhibition via FOXO/c-Myc Axis. Cancer Cell.

[29] Wang N (2018). MALAT1 promotes cisplatin resistance in cervical cancer by activating the PI3K/AKT pathway. Eur. Rev. Med. Pharm. Sci..

[30] Eisenhauer EA (2009). New response evaluation criteria in solid tumours: revised RECIST guideline (version 1.1). Eur. J. Cancer.

[31] Cibulskis K (2013). Sensitive detection of somatic point mutations in impure and heterogeneous cancer samples. Nat. Biotechnol..

[32] Li H, Durbin R (2009). Fast and accurate short read alignment with Burrows-Wheeler transform. Bioinformatics.

[33] Mermel CH (2011). GISTIC2.0 facilitates sensitive and confident localization of the targets of focal somatic copy-number alteration in human cancers. Genome Biol..

[34] Reardon B. (2018) Calculate Mutation Burden. http brendanreardon/calculate_mutational_burden (accessed 19 Mar 2021).

