# Supplementary material for: Phase I study of LZM005 in patients with HER2-positive metastatic breast cancer
Source: NPJ Breast Cancer. 2022 Dec 27;8:132. doi: 10.1038/s41523-022-00501-2 (PMC9794829; doi:10.1038/s41523-022-00501-2)
Supplement: Supplementary file 1 — reporting-summary [file 41523_2022_501_MOESM1_ESM.pdf]

nature portfolio

Corresponding author(s): Su Li; Yanxia Shi

Last updated by author(s): Nov 17, 2022

Reporting Summary

Nature Portfolio wishes to improve the reproducibility of the work that we publish. This form provides structure for consistency and transparency in reporting. For further information on Nature Portfolio policies, see our Editorial Policies and the Editorial Policy Checklist.

Statistics

For all statistical analyses, confirm that the following items are present in the figure legend, table legend, main text, or Methods section.

n/a	Confirmed	
☐	☒	The exact sample size (*n*) for each experimental group/condition, given as a discrete number and unit of measurement
☐	☒	A statement on whether measurements were taken from distinct samples or whether the same sample was measured repeatedly
☐	☒	The statistical test(s) used AND whether they are one- or two-sided<br>*Only common tests should be described solely by name; describe more complex techniques in the Methods section.*
☐	☒	A description of all covariates tested
☐	☒	A description of any assumptions or corrections, such as tests of normality and adjustment for multiple comparisons
☐	☒	A full description of the statistical parameters including central tendency (e.g. means) or other basic estimates (e.g. regression coefficient) AND variation (e.g. standard deviation) or associated estimates of uncertainty (e.g. confidence intervals)
☐	☒	For null hypothesis testing, the test statistic (e.g. *F*, *t*, *r*) with confidence intervals, effect sizes, degrees of freedom and *P* value noted<br>*Give P values as exact values whenever suitable.*
☒	☐	For Bayesian analysis, information on the choice of priors and Markov chain Monte Carlo settings
☒	☐	For hierarchical and complex designs, identification of the appropriate level for tests and full reporting of outcomes
☐	☒	Estimates of effect sizes (e.g. Cohen's *d*, Pearson's *r*), indicating how they were calculated

Our web collection on statistics for biologists contains articles on many of the points above.

Software and code

Policy information about availability of computer code

Data collection	Agilent Sure Select Human All Exon V6 Kit, ELLSA,
Data analysis	bwa, samblaster, Mutect2, GISTIC2, R 3.6.0,Phoenix WinNonlin 8.1

For manuscripts utilizing custom algorithms or software that are central to the research but not yet described in published literature, software must be made available to editors and reviewers. We strongly encourage code deposition in a community repository (e.g. GitHub). See the Nature Portfolio guidelines for submitting code & software for further information.

Data

Policy information about availability of data

All manuscripts must include a data availability statement. This statement should provide the following information, where applicable:

- Accession codes, unique identifiers, or web links for publicly available datasets
- A description of any restrictions on data availability
- For clinical datasets or third party data, please ensure that the statement adheres to our policy

he raw sequencing data were deposited at Sequence Read Archive database (PRJNA898328). Other data generated in this study were deposited at Research Data Deposit (https://www.researchdata.org.cn/, RDDA2022952814). If a researcher wants to use our raw data for scientific research purposes, he or she could apply for use with our corresponding author and database administrator.

Human research participants

Policy information about studies involving human research participants and Sex and Gender in Research.

Reporting on sex and gender	The participants in this trial all are women.
Population characteristics	In phase Ia,<br>14 female patients (median age, 46.5 years; range, 29 to 67 years) .<br>Ten patients (71.4%) received more than three lines of chemotherapy regimens in the metastatic period.<br>Twelve (85.7%) received prior trastuzumab treatment, including three patients in the adjuvant setting and 12 in the metastatic setting.<br>Ten patients (71.4%) presented with visceral metastasis, and five (35.7%) had liver metastasis.<br><br>In phase Ib,<br>20 female patients (median age, 52 years; range, 33 to 69 years) .<br>The mean number of previous chemotherapy regimens in the metastatic period was 1 (range, 0-5). Fourteen (70.0%) received prior trastuzumab treatment, including seven patients in the adjuvant setting, and 13 in the metastatic setting.<br>Sixteen patients (80.0%) showed visceral metastasis, and eight (40.0%) showed liver metastasis.
Recruitment	Patients who satisfied the following inclusion criteria were eligible for phases Ia and Ib:<br>1) age between 18 and 70 years,<br>2) a histologic diagnosis of MBC with a positive HER2 status (immunohistochemistry 3+, or 2+ confirmed by fluorescence in situ hybridization [FISH]). Hormone receptor status should be recorded but unrestricted.<br>3) an Eastern Cooperative Oncology Group [ECOG] score of 0 to 1,<br>4) presence of at least one measurable lesion according to the RECIST 1.1 criteria,<br>5) nonavailability of standard treatments or unwillingness to receive standard treatment,<br>6) adequate bone marrow and organ function,<br>7) left ventricular ejection fraction of 50% or more at baseline (determined by echocardiography).<br>There was no limit on the number of prior therapies.<br><br>Written informed consent was obtained from all patients before their enrollment in this study.
Ethics oversight	The study protocol was approved by the institutional review boards of Sun Yat-Sen University Cancer Center, Sun Yat-Sen Memorial Hospital (S.Y.S.M.H.), and Sun Yat-Sen First Affiliated Hospital (S.Y.S.F.A.H.; S.Y.S.M.H. and S.Y.S.F.A.H. participated in the phase Ib study).

Note that full information on the approval of the study protocol must also be provided in the manuscript.

Field-specific reporting

Please select the one below that is the best fit for your research. If you are not sure, read the appropriate sections before making your selection.

☒ Life sciences ☐ Behavioural & social sciences ☐ Ecological, evolutionary & environmental sciences

For a reference copy of the document with all sections, see nature.com/documents/nr-reporting-summary-flat.pdf

Life sciences study design

All studies must disclose on these points even when the disclosure is negative.

Sample size	No sample size predetermination was performed since this was the first in human phase I trial.
Data exclusions	No data were excluded.
Replication	The pre-clinical data were repeated with similar results 2- or 3 times.
Randomization	There was no randomization in this phase I, single are trial.
Blinding	There was no blinding in this phase I , single arm trial.

Reporting for specific materials, systems and methods

We require information from authors about some types of materials, experimental systems and methods used in many studies. Here, indicate whether each material, system or method listed is relevant to your study. If you are not sure if a list item applies to your research, read the appropriate section before selecting a response.

March 2021

Materials & experimental systems

n/a	Involved in the study
☒	☐ Antibodies
☒	☐ Eukaryotic cell lines
☒	☐ Palaeontology and archaeology
☒	☐ Animals and other organisms
☐	☒ Clinical data
☒	☐ Dual use research of concern

Methods

n/a	Involved in the study
☒	☐ ChIP-seq
☒	☐ Flow cytometry
☒	☐ MRI-based neuroimaging

Clinical data

Policy information about clinical studies

All manuscripts should comply with the ICMJE guidelines for publication of clinical research and a completed CONSORT checklist must be included with all submissions.

Clinical trial registration	The clinical trial numbers were CTR20170991 (phase Ia, 2017-09-11) and CTR20191921 (phase Ib, 2019-09-29), and the trials were registered in www.chinadrugtrials.org.cn.
Study protocol	The full protocol can be accessed at chinadurgtrials.org.cn OR email the corresponding author with appropriate purpose.
Data collection	The data were collected via hospital history system and investigator assistant at Sun Yat-Sen University Cancer Center, Sun Yat-Sen Memorial Hospital (S.Y.S.M.H.), and Sun Yat-Sen First Affiliated Hospital (S.Y.S.F.A.H.; S.Y.S.M.H. and S.Y.S.F.A.H. participated in the phase Ib study).
Outcomes	The primary endpoints included safety, tolerability, and the dose for further studies. The secondary endpoints were PK, immunogenicity response, and biomarker analysis.
