# Supplementary material for: Phase I study of LZM005 in patients with HER2-positive metastatic breast cancer
Source: NPJ Breast Cancer. 2022 Dec 27;8:132. doi: 10.1038/s41523-022-00501-2 (PMC9794829; doi:10.1038/s41523-022-00501-2)
Supplement: Supplementary file 2 — supplementary information [file 41523_2022_501_MOESM2_ESM.pdf]

supplementary information

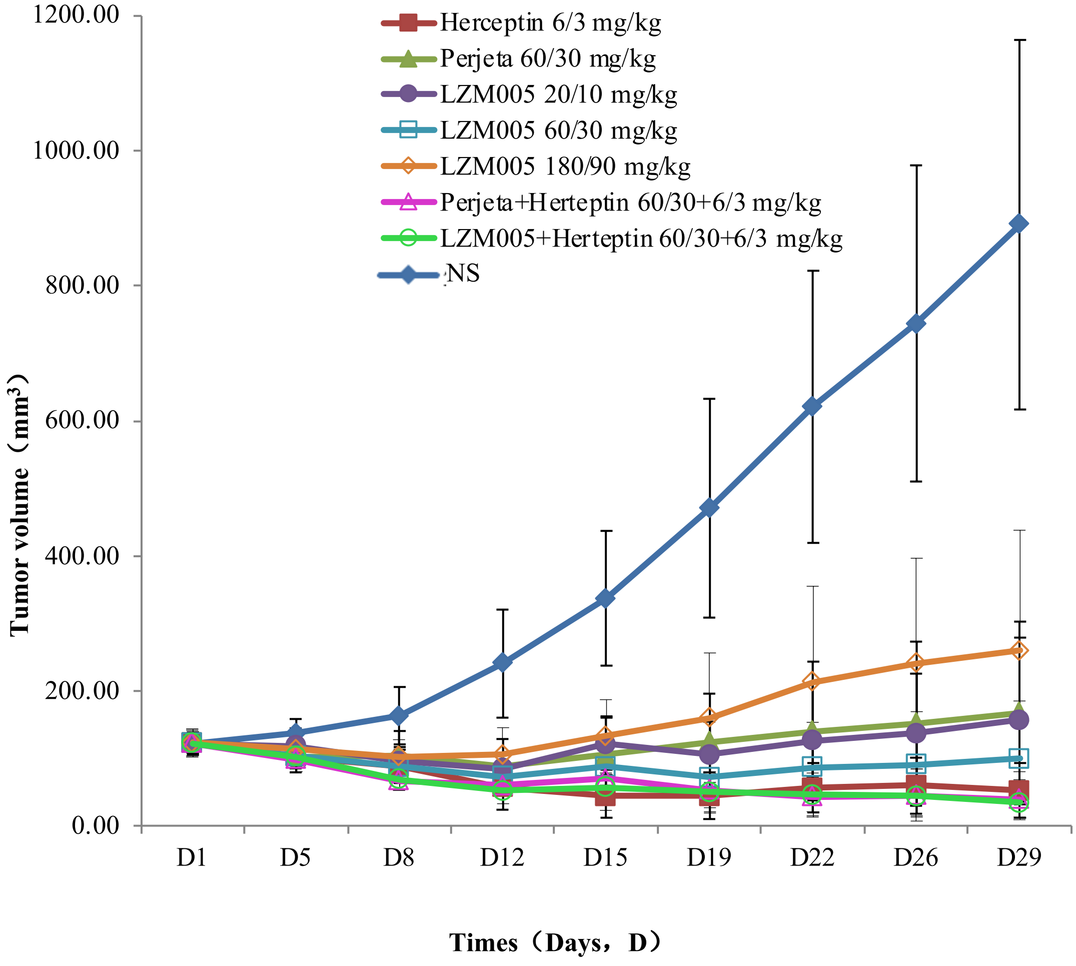

Supplementary Figure 1: The preclinical efficacy of LZM005.

Supplementary Table 1: The influences of genome alteration on durable clinical benefit

	Mutation Rate				
Symbol	Poor Response (N = 11)	Good Response (N = 7)	OR	*P*	*P*.adj
BRWD1	64%	43%	13.52	0.025	0.738
MELK	64%	43%	13.52	0.025	0.738
MIR4475	64%	43%	13.52	0.025	0.738
PCNT	64%	43%	13.52	0.025	0.738
RNF38	64%	43%	13.52	0.025	0.738
ASPA	73%	57%	11.13	0.031	0.738
TRPV1	73%	57%	11.13	0.031	0.738
TRPV3	73%	57%	11.13	0.031	0.738
KMT2B	9%	71%	0.16	0.152	0.738
